# Overstated Claims of Efficacy and Safety. Comment On: “Optimal Nutritional Status for a Well-Functioning Immune System Is an Important Factor to Protect Against Viral Infections”. *Nutrients* 2020, *12*, 1181

**DOI:** 10.3390/nu12092690

**Published:** 2020-09-03

**Authors:** Colby J. Vorland, Michelle M. Bohan Brown, Theodore K. Kyle, Andrew W. Brown

**Affiliations:** 1 Department of Applied Health Science, Indiana University School of Public Health-Bloomington, Bloomington, IN 47405, USA; 2 Independent Scientist, Bloomington, IN 47405, USA; mbohanb@iu.edu; 3 ConscienHealth, Pittsburgh, PA 15241, USA; Ted.Kyle@ConscienHealth.org

**Keywords:** COVID-19, spin, supplementation

Calder et al. published a narrative review on the role of various nutrients in immune function, with reference to COVID-19 [1]. The review promotes the authors’ recommendations of “safe and effective” nutrients to supplement for the general population for purported benefit, some of which exceed the recommended dietary allowance (RDA). To develop recommendations in a public health emergency, it is imperative to rely on quality systematic reviews of clinical evidence. We assessed some of the systematic reviews that the authors cite and the strength of some of the research is misrepresented to support these recommendations. We briefly outline examples here and provide additional quotations and information supporting our critiques on PubPeer (https://pubpeer.com/publications/850AC9189DFE4DFF70AB9CF9548F3B).

## 1. Example 1

Calder et al. write about the effects of nutritional formulas rich in antioxidants or eicosapentaenoic acid (EPA) and docosahexaenoic acid (DHA) on acute respiratory distress syndrome: “A recent Cochrane review of these trials identified a significant improvement in blood oxygenation and significant reductions in ventilation requirement, new organ failures, length of stay in the intensive care unit and mortality at 28 days”, citing [2]. However, the review concluded “little or no difference” in all-cause mortality from low quality evidence and “very low quality of evidence” for other outcomes, yielding uncertain benefit [2].

## 2. Example 2

Calder et al. recommend that “healthy individuals” supplement vitamin C for upper and lower respiratory tract infections, by referencing [3,4,5]. The first two references explicitly conclude that the evidence does not justify supplementation in the general population [3,4]. In [5], the authors repeatedly commit a unit-of-analysis error by pooling two arms of the same study, which invalidates the effect estimates.

## 3. Safety

Finally, safety is not guaranteed for nutrients, given the known issues with intakes above tolerable upper intake levels (UL). Adverse events were inadequately assessed in the context of purported benefits. Calder et al.’s recommended intakes do not adequately define amounts (supplement only, or total daily intake from all sources) or intended population given differences in UL by age/sex (e.g., their zinc recommendation is above the UL for children ≤ 3 y). The systematic review of omega-3 fatty acids and antioxidants that they cited could not rule out adverse events [2].

Though recommendations by Calder et al. are made in the context of the COVID-19 pandemic, we note that this review offers no direct evidence to support the claim for dietary supplements for prevention or treatment of COVID-19, per se [1]. The “absence of evidence of an effect” is not “evidence of absence of an effect”, and we thus acknowledge the possibility that better and longer studies may eventually support such recommendations. Similarly, the “absence of evidence of harm” should not be misinterpreted as “evidence of absence of harm” or evidence of safety. Despite the fact we only reviewed a subset of the articles cited by Calder et al., the appropriate consideration of the articles which we re-evaluated herein substantially alters the interpretation of the clinical evidence that they advanced. Thus, in the absence of strong evidence for or against supplementation, such recommendations for respiratory infections are unwarranted. 
